# What Goes around Comes around-A Comparative Study of the Influence of Chemical Modifications on the Antimicrobial Properties of Small Cyclic Peptides

**DOI:** 10.3390/ph6091130

**Published:** 2013-09-06

**Authors:** Kathi Scheinpflug, Heike Nikolenko, Igor V. Komarov, Marina Rautenbach, Margitta Dathe

**Affiliations:** 1 Leibniz-Institut für Molekulare Pharmakologie, Robert-Roessle-Str. 10, Berlin 13125, Germany; E-Mails: scheinpflug@fmp-berlin.de (K.S.); nikolenko@fmp-berlin.de (H.N.); 2 Institute of High Technologies, Kiev National Taras Shevchenko University, 64 Vladimirskaya st., Kiev 01033, Ukraine; E-Mail: ik214@yahoo.com; 3 Department of Biochemistry, University of Stellenbosch, Private Bag X1, Matieland 7602, South Africa; E-Mail: mra@sun.ac.za

**Keywords:** cyclic hexapeptides, antimicrobial activity, mode of action, permeabilisation, chemical modification

## Abstract

Tryptophan and arginine-rich cyclic hexapeptides of the type cyclo-RRRWFW combine high antibacterial activity with rapid cell killing kinetics, but show low toxicity in human cell lines. The peptides fulfil the structural requirements for membrane interaction such as high amphipathicity and cationic charge, but membrane permeabilisation, which is the most common mode of action of antimicrobial peptides (AMPs), could not be observed. Our current studies focus on elucidating a putative membrane translocation mechanism whereupon the peptides might interfere with intracellular processes. These investigations require particular analytical tools: fluorescent analogues and peptides bearing appropriate reactive groups were synthesized and characterized in order to be used in confocal laser scanning microscopy and HPLC analysis. We found that minimal changes in both the cationic and hydrophobic domain of the peptides in most cases led to significant reduction of antimicrobial activity and/or changes in the mode of action. However, we were able to identify two modified peptides which exhibited properties similar to those of the cyclic parent hexapeptide and are suitable for subsequent studies on membrane translocation and uptake into bacterial cells.

## 1. Introduction

Antimicrobial peptides (AMPs) are ubiquitous defence molecules in organisms and have been shown to exhibit promising potential as a new class of antibiotics [1]. Among the structurally diverse AMPs, short sequences rich in arginine (R) and tryptophan (W) are of particular interest as cationic charge and amphipathicity represent the structural prerequisite for an initial electrostatic interaction of the peptide with negatively charged lipid systems and subsequent insertion into the lipid membrane [2].

The most common antimicrobial mechanism of action can be described by different models of membrane permeabilisation. Here, interaction with the lipid bilayer results in loss of membrane integrity, e.g., by pore formation, but also detergent-like peptide properties have been reported [3]. In recent years, research on antimicrobial activity has led to the conclusion that there are also some cases where membrane permeabilisation is neither the only nor the dominating mode of action [4,5]. Translocation of AMPs into the cytoplasm could be observed, where the peptides interfere with cellular processes, such as protein synthesis and DNA replication. In addition, growing evidence suggests peptide interaction also with functional domains within the membrane which might result in lipid demixing and altered protein activity [6].

We could show that the synthetic cyclic hexapeptide cWFW with the sequence c-RRRWFW has an increased antibacterial activity against Gram-positive and Gram-negative bacteria compared to the linear sequence, while no toxicity towards eukaryotic cells could be detected [7]. Despite its pronounced antimicrobial potential, investigations on the mechanism of action revealed that cWFW does not act by the membrane permeabilisation mechanism generally associated with AMPs [8]. Recent studies of a set of K-/R- and W-rich linear peptides indicated that several of these compounds were able to penetrate the cell membrane of *E. coli* and to accumulate in the cytoplasm [9]. Furthermore, cyclisation-induced enhanced backbone rigidity has been suggested to increase the uptake efficiency of R-rich peptides for eukaryotic cells [10].

In order to examine putative translocation into the cytoplasm of bacterial cells, our hexapeptide had to be modified according to the requirements of the particular analytical techniques. For analysis with confocal laser scanning microscopy (CLSM) fluorescent analogues labeled with carboxyfluorescein (Fluos), coumarin (Cu) or nitrobenzoxadiazole (NBD) were synthesized. Furthermore, peptide uptake studies using an HPLC-based strategy developed by Oehlke *et al*. [11] required the introduction of free amino groups for chemical modification.

In the present study we characterized the feasibility of the cyclic hexapeptide analogues for the above investigations by examining the consequences of peptide modification according to antibacterial activity and mechanism of action.

## 2. Experimental Section

### 2.1. Materials

Components for peptide synthesis were purchased/prepared as follows: Fmoc-Arg(Pbf)-OH, Fmoc-Lys(Boc)-OH, Fmoc-Trp(Boc)-OH (GL Biochem, Shanghai Ltd., Shanghai, China), Fmoc-L-Dap(Dde)-OH (Iris Biotech, Marktredwitz, Germany), 4-Chloro-7-nitro-1,2,3-benzoxadiazole, (NBD-Cl, Sigma-Aldrich, St. Louis, MO, USA), 6-bromo-7-hydroxycoumarin-4-yl [12]. Chemical components for buffer preparation were purchased from Fluka (Taufkirchen, Germany). Triton X-100, EtOH and HCl came from Merck (Darmstadt, Germany). Materials for peptide characterisation were: *E. coli* DH5α, *B. subtilis* DSM 347, HeLa S (all from DSMZ, Braunschweig, Germany), human erythrocyte concentrate (Charité - Universitätsmedizin Berlin, Berlin, Germany), Gibco^®^ Dulbecco’s Modified Eagle Medium (DMEM), Gibco^®^ Dulbecco’s Phosphate Buffered Saline (DPBS), L-glutamine and penicillin-streptomycin (pen-strep) (all Life Technologies Corp., Darmstadt, Germany), fetal calf serum (FCS, Biochrom, Berlin, Germany), D-glucose (Sigma-Aldrich), lysogeny broth (LB, Sigma-Aldrich), nitroaniline (Sigma-Aldrich), 1-palmitoyl-2-oleoylphosphatidyl-sn-glycerol (POPG; Avanti Polar Lipids, Inc., Alabaster, AL, USA), polymyxin sulfate B (PMX, Fluka), propidium iodide (PI), sodium dodecyl sulfate (SDS) and sodium nitrite (all Sigma-Aldrich). Consumables used in cell culture were obtained from TPP (Trasadingen, Switzerland). FACS solutions came from Becton Dickinson (Heidelberg, Germany). For HPLC analysis: trifluoroacetic acid (TFA, Acros Organics, Geel, Belgium) and acetonitrile (VWR Chemicals, Darmstadt, Germany) were used.

### 2.2. Peptide Synthesis

The fluorescence-labeled peptides cW_2_[Fluos], cW_3_[Fluos] and cW[Cu]W (for sequences refer to (Table 1) were provided by Biosyntan (Berlin, Germany). The synthesis of the parent peptide cWFW and the lysine-containing analogues has been described previously [13]. Peptides were prepared by multiple solid phase synthesis using Fmoc/tBu strategy according to SHEPPARD [14]. Cleavage from resin and removal of protecting groups was done as described before [15], and cyclization was achieved manually by HAPyU chemistry [16]. Peptide purification and analysis were performed by high performance liquid chromatography (HPLC) on a Jasco LC-2000Plus (Tokyo, Japan) and Dionex UltiMate 3000 with ProntoSil 300-5-C18-H columns (250 × 4.6 mm, 5 µm) (Bischoff Chromatography, Leonberg, Germany). Peptide mass was determined by UPLC-MS (ultra-performance liquid chromatography mass spectrometry) on an ACQUITY UPLC^®^ System by Waters (Milford, MA, USA) using an Ascentis^®^ Express Peptide ES-C18 column (3 × 2.1 mm, 2.7 µm) (Sigma Aldrich). Final peptide purity was determined to be >95%.

### 2.3. CD Spectroscopy

Cyclic hexapeptides were dissolved to 100 µM in phosphate buffer (10 mM NaH_2_PO_4_/Na_2_HPO_4_, 154 mM NaF, pH 7.4). To mimic a membrane-like environment, SDS or POPG SUVs (small unilamellar vesicles) were added to give final concentrations of 25 mM detergent and 10 mM lipid. Due to light scattering of liposomes, spectra could only be recorded down to 205 nm. Vesicle preparation has been described previously [17]. Dried POPG lipids were dissolved in phosphate buffer and sonicated on ice for 20 min. CD spectra were obtained on a Jasco 720 spectrometer (Japan). Twenty scans were accumulated within the range of 260 nm and 190 nm using a 2 mm pathlength quartz cell. Results are presented as mean residue molar ellipticity Θ_mr_.

### 2.4. Hydrophobicity

The retention time *t*_R_ of the cyclic hexapeptides was determined by HPLC on a Jasco LC-2000Plus using a ProntoSil 300-5-C18-H column (250 × 4.6 mm, 5 µm) (Bischoff Chromatography). Peptide concentration was 1 mg/mL in mobile phase A (0.1% TFA in deionized water), mobile phase B consisted of 0.1% TFA in 80% acetonitrile in deionized water. For chromatographic analyses a linear gradient of 5%–95% phase B over 40 min at 22 °C was applied.

### 2.5. Antimicrobial Activity

The minimal inhibitory concentration (MIC) of the cyclic hexapeptides was tested against Gram-negative *E. coli* DH5α and Gram-positive *B. subtilis* DSM 347 using a microdilution technique in 96 well microtiter plates as described previously [18]. Briefly, cells from an overnight culture were inoculated 1:100 in LB medium, grown to mid log phase (OD_600_ 0.4 ± 0.1) and adjusted to 5 × 10^5^ cells/well. Final peptide concentrations ranged from 0.05 µM to 100 µM (2-fold dilutions) and were tested in triplicates in at least three independent experiments. The MIC was determined after 18 h of incubation at 37 °C, 180 rpm being the lowest peptide concentration at which no visible bacterial growth can be detected photometrically (A_600_, Safire Microplate Reader, Tecan, Männedorf, Germany).

### 2.6. Hemolytic Activity

The lytic effect of selected peptides upon human erythrocytes was determined as described before [7]. Briefly, cells were washed thoroughly in Tris buffer (10 mM Tris, 150 mM NaCl, pH 7.4). Suspensions with 2.5 × 10^8^ cells/mL were incubated with peptides at a final concentration of 100 µM for 30 min at 37 °C. After centrifugation, 0.6% NH_4_OH was added to an aliquot of the supernatant and hemoglobin absorption (A) was measured at 540 nm (Jasco V-550). Whole cell suspensions exposed to NH_4_OH were used as positive control (A_100%_) and preparations without peptide served as negative control (A_0%_), respectively. Data were collected in three independent experiments.

### 2.7. Propidium Iodide Influx

Membrane permeabilisation was determined by fluorescence activated cell sorting (FACS) on a FACSCalibur (Becton Dickinson) equipped with a 488 nm argon laser. Propidium iodide (PI, 10 µg/mL) and peptides at a final concentration corresponding to the respective MIC were dissolved in LB. Cells were grown to mid log phase (OD_600_ 0.4 ± 0.1) and added to the solution just before the measurement at a concentration of 5 × 10^5^ cells/mL. Cells were sorted on the basis of PI fluorescence which increases upon intercalation with DNA. This can only be observed in the case of disturbed membrane integrity as PI cannot translocate across intact lipid bilayers. Cell fluorescence was measured after incubation time points of 0, 5, 10, 15, 20, 30, 60 and 90 min at 37 °C, 180 rpm. A minimum of 20,000 cells was recorded for each preparation. CellQuest^TM^ Pro software (Becton Dickinson) was used for data acquisition and results were analyzed with FCS Express V3 (De Novo Software, Los Angeles, CA, USA).

### 2.8. Cellular Uptake

The applicability of chemically modified peptides in uptake experiments was tested with cR2[Cu] into HeLa S cells. The well characterized cell-penetrating peptide penetratin [19], here labeled with carboxyfluorescein (Fluos-penetratin), was used as positive control. HeLa S cells were cultivated in DMEM supplemented with 1 g/L D-glucose, 1% FCS and 1% pen-strep. For uptake experiments, cells were harvested and diluted to 1 × 10^6^ cells/mL in PBS supplemented with 1 g/L D-glucose. Peptides were added at final concentrations of 18 µM (cR2[Cu]) and 9 µM (Fluos-penetratin), respectively, and cells were incubated at 37 °C for 1 h with careful shaking (Thermomixer Comfort, Eppendorf, Wesseling-Berzdorf, Germany). After centrifugation (1,500 × *g*, 4 min, 0 °C, Biofuge primoR, Heraeus, Bremen, Germany), the supernatant was removed and stored for subsequent HPLC studies to quantify peptide amount which has not been accumulated or internalized. Cells were washed twice with ice-cold PBS and finally resuspended in 0.5 mL ice-cold PBS. As described previously, 10 µL of a freshly prepared diazotized 2-nitroaniline solution was added and allowed to react for 10 min at 0 °C (ice bath) in order to modify membrane surface bound peptides [11,20]. After repeated washing with ice-cold PBS, cells were lysed with 500 µL 0.1% Triton X-100/0.1% TFA and stored at −20 °C until further use. Before HPLC analysis lysates were thawed at room temperature. BCA^TM^ Protein Assay Kit (Thermo Scientific, Bremen, Germany) was used for protein determination. The average protein content of the lysates of 10^6^ cells was found to be 130 µg. Three independent experiments were performed in triplicate.

The non-modified, translocated peptide was analysed by HPLC on a Jasco LC-2000Plus using a ProntoSil 300-5-C18-H column (250 × 4.6 mm, 5 µm) (Bischoff Chromatography) and a precolumn with PolyenCap A300, 10 µm. Up to 200 µL cell lysate were loaded onto the column. Peptide elution was realized with 0.1% TFA/deionized water (A) and 0.1% TFA in 80% acetonitrile/deionized water (B) at a flow rate of 1 mL/min and a gradient of 20%–70% B over 20 min. Quantification was performed by fluorescence measurement of carboxyfluorescein (ex 445 nm, em 520 nm) and methoxycoumarin (ex 350 nm, em 385 nm). Data were calibrated with standard solutions containing pure hexapeptide and Fluos-penetratin in 0.1% TFA (A) prepared under identical conditions.

## 3. Results and Discussion

### 3.1. Peptide Modifications and Antimicrobial Activity

An overview of the antimicrobial peptides used in this study is given in Table 1. The cyclic hexapeptide cWFW served as template for the chemical modifications necessary for further peptide investigation.

**Figure 1:**
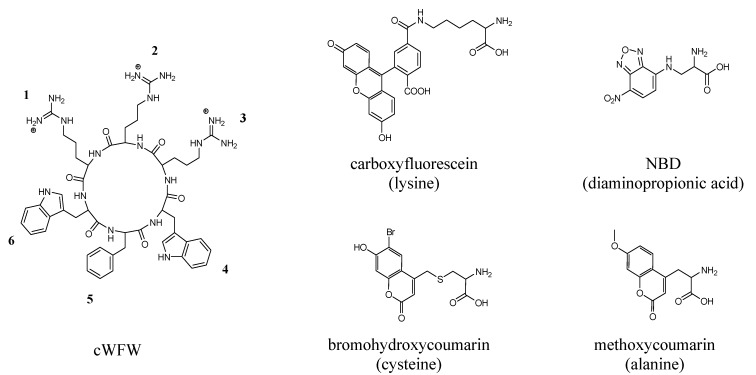
Chemical structures of cWFW and the four fluorescent labels used in this study. Fluorophores are depicted coupled to the respective amino acid (latter given in brackets) used to introduce the labels into the peptide ring.

Confocal laser scanning microscopy (CLSM) requires the introduction of fluorescent labels (Figure 1). The successful application of carboxyfluorescein (Fluos)-labeled peptides in uptake studies is well documented [9,13,21]. Coumarin (Cu) and nitrobenzoxadiazole (NBD) were chosen due to their hydrophobic nature and structural similarity to tryptophan. The chemical derivatives methoxycoumarin and bromohydroxycoumarin (BHCM) differ in their fluorescence maxima with 396 nm and 468 nm, respectively. BHCM has been reported as photoactivatable protecting group with high quantum yield and increased solubility in aqueous solutions [12].

The HPLC approach to investigate peptide translocation is based on the chemical modification of extracellular and surface-bound peptides by diazotized 2-nitroaniline [20]. After cell lysis, non-modified (internalized) and nitroaniline-exposed peptide moieties can be distinguished by HPLC separation. As the chemical modification reaction is restricted to free amino groups, arginine was substituted by lysine residues at different positions in the peptide ring.

All peptides (except cKRK) were more active towards the Gram-positive *B. subtilis* than the Gram-negative *E. coli*, thus confirming the activity profile of this class of peptides [7,22,23]. However, all modifications applied to cWFW led to an overall reduction in antimicrobial activity (Table 1). The bulky carboxyfluorescein, introduced via a lysine linker into the hydrophobic (cW_2_[Fluos]) as well as polar domain (cW_3_[Fluos]), led to a loss in positive net charge compared to the parent peptide. Both, the large size of the Fluos-fluorophore, as well as the change in hydrophobicity and amphipathicity might be responsible for the pronounced loss of antimicrobial activity to a level which was not observed for any of the other fluorescent labeled peptides (Table 1).

As small modifications at the central position of the hydrophobic WFW cluster were shown to exhibit the least influence on antimicrobial activity [8], coumarin and NBD were introduced to substitute phenylalanine in order to maintain the overall amphipathic character of the molecule. Nevertheless, both labels also reduced peptide antimicrobial activity and in most cases led to an increase in hemolytic activity. Structural differences between the coumarin derivatives generally did not influence the antibacterial effect of cW[Cu]W and cW[S-Cu]W.

The decrease in antibacterial activity observed after substitution of arginine residues with lysine in the polar cluster of the cyclic hexapeptides was slightly more pronounced compared to the decrease in activity found for the fluorescent labeled peptides (excluding the Fluos-labeled peptides) (Table 1). Of the lysine-containing peptides, cKRK showed the best antibacterial potential. One arginine located in the centre of the cationic sequence motif seems to be highly important for optimal peptide interaction with bacterial membranes. The K-analogue cRKK exhibited significant hemolytic activity.

The peptides cR2[Cu], cR2[S-Cu] and cR2[NBD] were synthesized to combine the high activity of cKRK with the best antibacterial properties found for the fluorescent analogues. The combination of both modifications had no additional negative influence on antimicrobial activity and kept the hemolytic activity low (Table 1). The most active peptides against the two bacterial strains bearing a fluorophore and two chemically reactive amino groups were cR2[Cu] and cR2[NBD]. To confirm if these analogues are suitable candidates for membrane translocation studies, the structural character and interaction with model membranes were determined and compared with that of the parent hexapeptide.

**Table 1:** Sequences, antimicrobial and hemolytic activity of the cylic hexapeptide analogues. Minimal inhibitory peptide concentrations (MIC) were determined in triplicates in at least three independent experiments. Hemolytic activity was determined as % hemolysis at a peptide concentration of 100 µM.

Peptides	Sequences	MIC [µM]		Hemolysis [%] at 100 µM
		*E. coli* DH5α	*B. subtilis* DSM 347	
cWFW	*c-RRRWFW*	3	3	4
cW_2_[Fluos]	*c-RRRWWK[Fluos]*	>100	>100	9
cW_3_[Fluos]	*c-RRWWWK[Fluos]*	>100	>100	2
cW[Cu]W	*c-RRRW[Ala-Coumarin]W*	25	6	23
cW[S-Cu]W	*c-RRRW[Cys-Br-Coumarin]W*	25	6	76
cW[NBD]W	*c-RRRW[Dap-NBD]W*	50	25	76
cRKR	*c-RKRWFW*	50	12	4
cRKK	*c-RKKWFW*	50	25	56
cKRK	*c-KRKWFW*	12	25	2
cKKR	*c-KKRWFW*	100	25	3
cKKK	*c-KKKWFW*	100	50	8
cR2[Cu]	*c-KRKW[Ala-Coumarin]W*	50	6	4
cR2[S-Cu]	*c-KRKW[Cys-Br-Coumarin]W*	50	25	5
cR2[NBD]	*c-KRKW[Dap-NBD]W*	50	12	26

Cu—coumarin; Dap—diaminopropionic acid; Fluos—carboxyfluorescein; NBD—nitrobenzoxadiazole.

### 3.2. Peptide Hydrophobicity and Conformation

**Figure 2:**
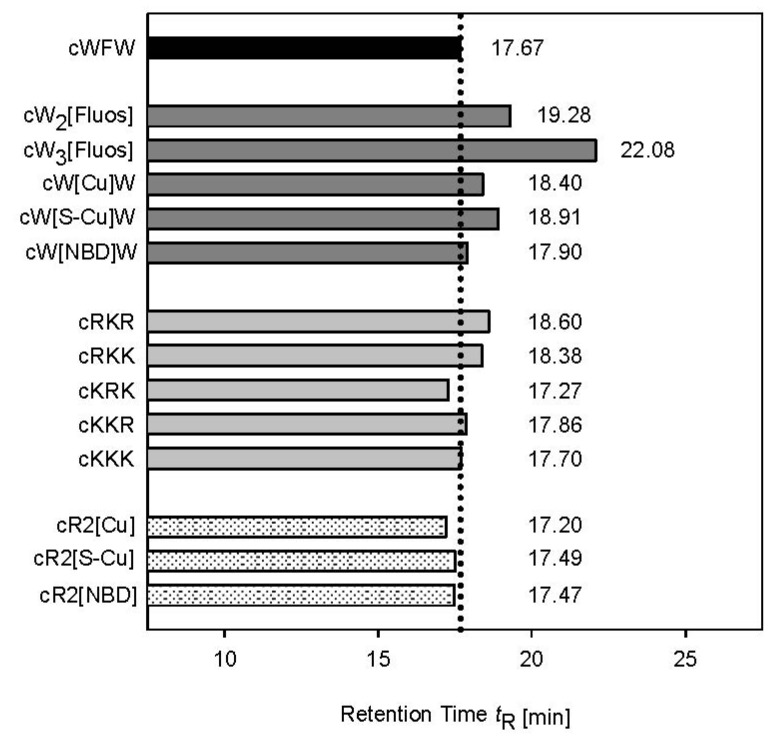
Retention times *t*_R_ of the cyclic hexapeptides. The influence of fluorescent labels (dark grey), lysine substitution (light grey) and double modifications (shaded) on interaction with the hydrophobic column is shown compared to the parent peptide cWFW (black).

To investigate the influence of fluorescent label modification and amino acid substitution on the hydrophobicity/amphipathicity of the cyclic hexapeptide derivatives, we determined the retention time *t*_R_ by reversed phase HPLC (Figure 2). The method monitors the retention of compounds by the hydrophobic HPLC stationary phase. Differences in *t*_R_ reflect differences in the effective hydrophobicity of the molecule which is related to the intrinsic hydrophobicity of the individual residues and their ability to form hydrophobic domains for interaction with the HPLC matrix. At the same time, hydrophilic residues will locate at the opposite side of the molecule. Reduction in the size or positioning of cationic residues next to the hydrophobic molecular surface area would reduce amphipathicity. *t*_R_-measurements have successfully been applied to characterize amphipathic helices [24], β-structured peptides [25] and different sets of our small cyclic R-, W-rich peptides [23,26,27].

The introduction of fluorophores generally increased retention time. This can be related to an enlargement of the aromatic ring system compared to the phenylalanine side chain with the consequence of improved interaction with the non-polar solid surface. The most pronounced increase in *t*_R_ was determined for cW_3_[Fluos]. The enhanced retention on the hydrophobic matrix, which was largely based on the reduction in overall charge in both carboxyfluorescein-labeled peptides, related to a decrease in antibacterial activity (Table 1, Figure 2). Also, most of the K-containing analogues showed slightly higher hydrophobicity than cWFW. Interestingly, cKRK, the only peptide displaying a retention time lower than that of the parent peptide, showed pronounced antimicrobial activity (Table 1). The strong influence of these particular lysine substitutions on the peptide’s properties is reflected in cR2[Cu], cR2[S-Cu] and cR2[NBD] which are also less hydrophobic compared to cWFW.

A positive correlation between antimicrobial activity and hydrophobicity/amphipathicity has been reported for a large number of helical membrane-permeabilising peptides [24,28]. A high hydrophobicity/amphipathicity allows deep peptide penetration and efficient disturbance of the lipid matrix of bacterial membranes. Studies with a set of diastereomers of the cyclic β-sheet forming gramicidin S analogue GS14 showed a different picture. Here, amphipathicity reflected by *t*_R_ positively correlated with hemolytic activity but was found to be highly disadvantageous for the antimicrobial activity of GS14 peptides. A pronounced amphipathic nature was suggested to be undesirable in the design of constrained cyclic antimicrobial peptides [25]. Furthermore, investigations on tyrocidines (cyclic decapeptides analogous to GS) also showed that high *t*_R_ values may reduce the antimicrobial activity and a fine amphipathic balance must be maintained in order for the molecules to remain active [29,30]. The negative correlation between *t*_R_ and antimicrobial activity found with our small cyclic peptides supports the idea that high hydrophobicity/amphipathicity is also not crucial for their antimicrobial action.

**Figure 3:**
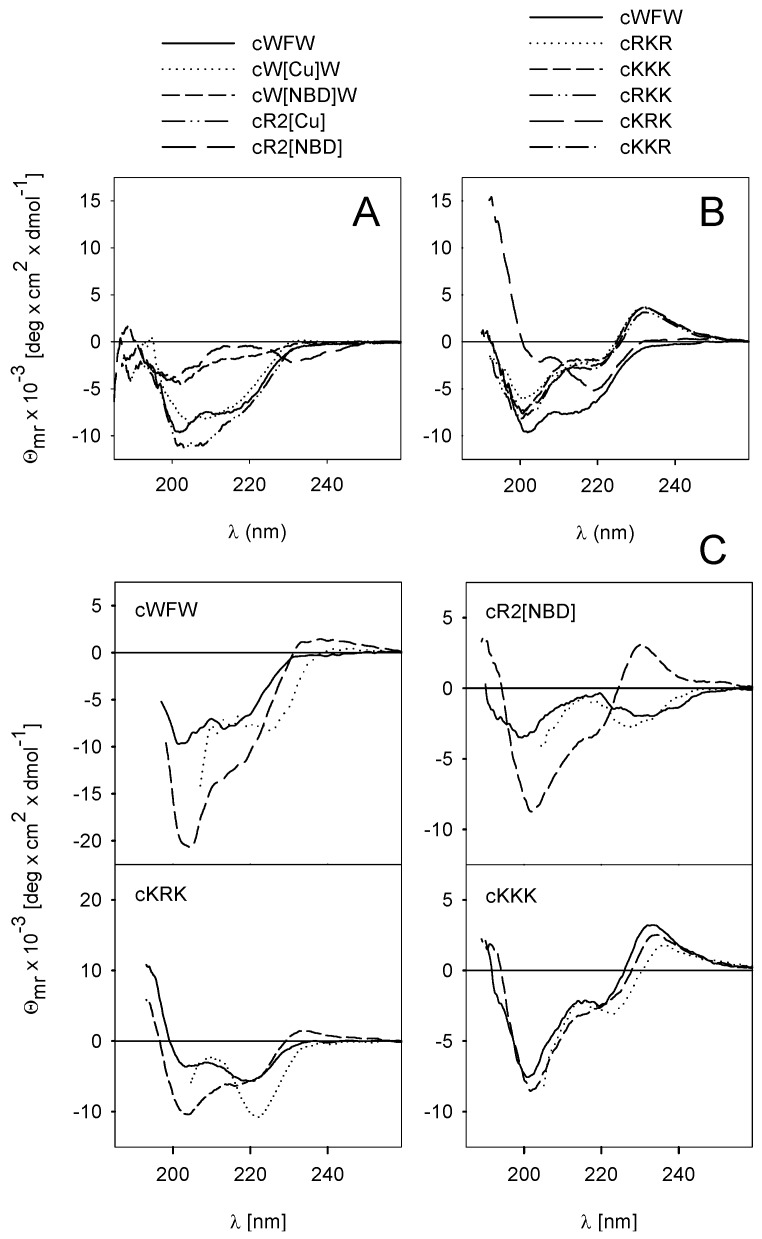
CD spectra of the cyclic hexapeptides in different solvent systems. (**A**) Lysine-substituted and (**B**) fluorescent-labeled peptides compared to cWFW in phosphate buffer. The effect of membrane-mimicking additives on the structure of selected lysine peptides is shown in (**C**): phosphate buffer (solid lines), 25 mM SDS (dashed lines) and 10 mM POPG-SUVs (dotted lines). Peptide concentration was 100 µM.

In order to get insight into the influence of chemical modifications on peptide conformation we performed CD spectroscopic studies (Figure 3). The spectrum of the parent peptide cWFW in buffer is characterized by a negative ellipticity minimum at 202 nm and a shoulder at 220 nm (Figure 3A, solid line). The CD spectra at the far UV wavelengths result from the backbone peptide bonds while contributions of both, peptide bonds and aromatic side chains, superimpose in the 220–230 nm region. The spectrum of cWFW is comparable to that of cyclo-RRWWRF (cRW) [7] which is characterized by two β-turns in the backbone and a rather flexible structure in aqueous solution [31]. Coumarin had only minor effects on the CD characteristics of the cyclic hexapeptide (Figure 3A, dotted line). However, the contribution of an NBD-side chain (cW[NBD]W), Figure 3A, short dash line) is related to a reduction of the negative ellipticity values. The intensity decrease and conservation of the ellipticity bands and shoulder positions could be associated with changes in backbone flexibility.

Among the K-containing peptides, cKRK showed the strongest shift in the spectrum with a high positive ellipticity below 200 nm (Figure 3B) which points to pronounced changes in backbone conformation. These changes are caused by a decrease in the effective hydrophobic surface area as reflected by a reduced retention time (Figure 2). In contrast, the other K-analogues are characterized by a positive ellipticity maximum at 230 nm, but minor spectral changes in the far UV wavelength range. This maximum is likely to result from a different orientation of the aromatic side chains relative to the peptide backbone [32], compared to cWFW and cKRK. Analysis of the spectra of cKRK, cW[NBD]W (Figure 3B, long dash line and 3A, short dash line) and cR2[NBD] (3A, long dash line) indicates that the fluorophore predominates the CD characteristics of the hexapeptide, which combines the polar KRK- with the hydrophobic W[NBD]W-cluster.

Both, detergent micelles and liposomes are often used in spectroscopic studies to mimic the anisotropic nature of the lipid matrix of cell membranes. Bound to micelles cWFW adopts an amphipathic structure with two β-turns [33]. All the aromatic residues point to the same direction establishing the hydrophobic part and the arginine side chains are arranged in a way to interact with the polar head groups on the micellar or liposomal surface. Thus, the intensity increase in ellipticity at 202 nm and the red shift of the shoulder at 220 nm to 225 nm, respectively, imply enhanced backbone rigidity and changes in the positioning of the aromatic residues upon partitioning of cWFW into SDS micelles and POPG liposomes (Figure 3C, top left column). Interestingly, the four peptides presented in Figure 3C with different antimicrobial activities (Table 1) and different CD spectra in buffer showed only minor conformational differences when bound to POPG (Figure 3C, dotted lines) and almost identical spectral characteristics in interaction with SDS micelles (Figure 3C, dashed lines). These observations underline the flexibility of most of the cyclic hexapeptides and their potential to adopt an amphipathic structure. Almost identical *t*_R_ values (Figure 2) point to comparable effective hydrophobic surface areas of the four peptides under appropriate conditions (Figure 3C). However, quite different antimicrobial activities support the hypothesis that binding to the lipid matrix of the bacterial membrane and peptide conformation may not be the major determinants of the biological effect.

### 3.3. Mode of Antimicrobial Action

**Figure 4:**
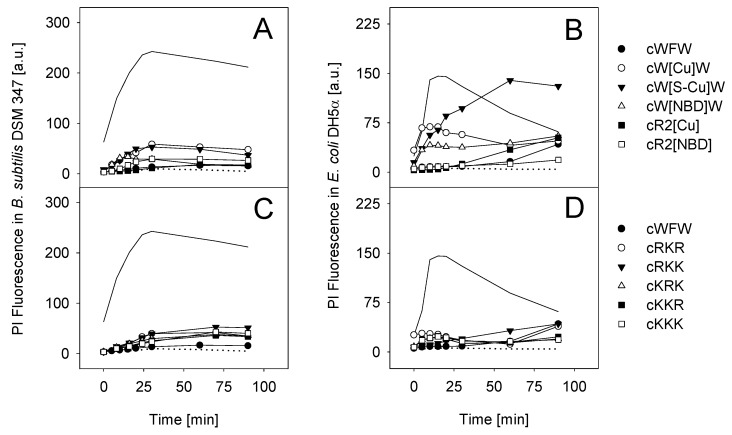
Bacterial membrane permeabilisation determined with flow cytometry using propidium iodide (PI). PI influx after incubation with cWFW, fluorescent-labeled and K-substituted cyclic hexapeptides was tested into *B. subtilis* DSM 347 (**A**,**C**) and *E. coli* DH5α (**B**,**D**) at respective MICs. The antibiotic polymyxin B (5 µM) and the helical model peptide KLA-1 (KLALKLALKALKAALKLA-NH_2_, 5 µM) obtain a high membrane permeabilising potential and served as positive control in Gram-negative and Gram-positive cells, respectively (solid line) [20,34]. Preparations without peptide were used as negative control (dotted line).

In order for the fluorescence-labeled and K-containing derivatives to be applicable in investigations on the mode of action of our cyclic hexapeptides, the chemical modifications must have no or only little influence on the properties of cWFW. As all peptide analogues used in this study fulfil the structural prerequisites for membrane interaction, *i.e.*, high amphipathicity and positive net charge, we investigated their potential to permeabilise the bacterial membrane (Figure 4).

All peptides induced no or only slight membrane permeabilisation when tested against *B. subtilis* (Figure 4, left column). Propidium iodide (PI) influx, due to membrane damage/permeabilisation, was slightly higher for cW[Cu]W and cW[S-Cu]W while cR2[Cu] and cR2[NBD] showed results similar to those found for cWFW. Lysine-substituted peptides had minor influence on the membrane integrity of Gram-positive as well as Gram-negative cells (Figure 4C,D). In contrast, treatment of *E. coli* with most of the fluorescent-labeled peptides resulted in pronounced membrane disruption with the order cW[S-Cu]W > cW[Cu]W > cW[NBD]W > cR2[Cu] (Figure 4B). No *E. coli* membrane permeabilisation could be detected for cR2[NBD].

As demonstrated for the cyclic hexapeptide cWFW [8], the investigated lysine analogues also appear to exert their antibacterial activity via a non-permeabilising mechanism. However, chemical modifications in the hydrophobic cluster by introducing fluorophores seem to change the mode of peptide interaction with the *E. coli* membrane. Here*,* membrane permeabilisation, as monitored by PI influx, correlated with peptide hydrophobicity (see Figure 2). An increase in hydrophobicity of this class of cyclic peptides and a large number of other antimicrobial sequences was reported to be linked to a deeper peptide insertion into and subsequent enhanced disturbance of the lipid matrix of cellular membranes [7,24,26].

With respect to the investigation of peptide translocation across bacterial membranes, cR2[Cu] and cR2[NBD] seem to be the most promising fluorescent-labeled cyclic hexapeptide candidates mimicking cWFW. Applying the established strategy for uptake studies in eukaryotic cells, described by Oehlke *et al*. [11], we tested the internalization of cR2[Cu] in comparison to carboxyfluorescein-labeled penetratin. Penetratin is a well characterized cell-penetrating peptide [35,36] which has been used for transmembrane delivery of a diversity of compounds [37,38].

**Figure 5:**
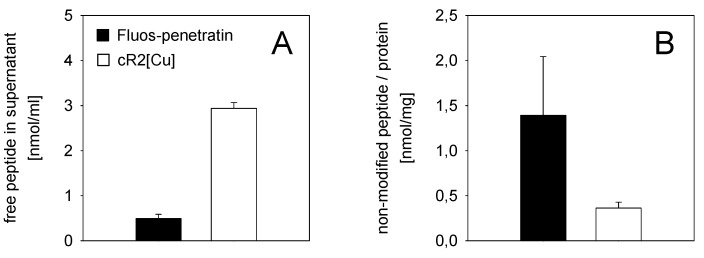
HPLC-based investigation of peptide uptake into HeLa S cells. (**A**) Amount of non-modified, free peptide in the supernatant after 1 h incubation at 37 °C with cR2[Cu] (18 nmol) and Fluos-penetratin (9 nmol), (**B**) non-modified peptide after extensive washing of cells and exposure to diazotized 2-nitroaniline. For each peptide, two independent experiments were performed in triplicates.

Figure 5A shows that a large amount of free cyclic peptide remained in the supernatant of the incubation solution after 1 h treatment of HeLa S cells, compared to Fluos-penetratin. At the same time, after chemical modification of cell surface located peptides, only little cR2[Cu] inaccessible to diazotized 2-nitroaniline could be detected, whereas a large amount of penetratin remained unmodified (Figure 5B).

The fraction of translocated penetratin showed to be comparable to the amount of other amphipathic peptides internalized into different cell lines [11,34,39], while the cellular uptake of the cyclic hexapeptide appeared to be low. The latter can be explained as cR2[Cu] also showed weak hemolytic activity, which confirms that this peptide, as the parent cWFW, does not interact well with the neutral membranes of eukaryotic cells. These experiments in eukaryotic cells, however, demonstrate the general applicability of the lysine- and fluorophore-bearing cyclic hexapeptides for fluorescence- and HPLC-based uptake studies.

## 4. Conclusions

Our research on cyclic antimicrobial peptides leads us to the conclusion that minor modifications within a small, structurally constrained peptide such as cWFW can result in unpredictable changes in antibacterial activity and mode of action. The clustered alignment of polar and hydrophobic amino acids in the peptide ring dictates the high level of amphipathicity which is necessary for peptide interaction with the bacterial membrane. Although the introduced modifications were chosen on the basis of the most suitable properties concerning size, hydrophobicity and net charge, the optimal interplay between arginine and the aromatic residues proved to underlie a delicate amphipathic balance. This study emphasized the fact that careful consideration needs to be taken when modifying small peptides to generate appropriate tools for biophysical and cellular studies especially when adding fluorophores. Our attempt to optimize the structure of the cyclic hexapeptides for fluorescence microscopy and HPLC-based studies to investigate uptake into bacterial cells resulted in the selection of c-KRKW[Dap-NBD]W and c-KRKW[Ala-Coumarin]W. In comparison to the parent peptide, these analogues displayed good antimicrobial activity and similar biophysical properties with regard to retention behavior and structure in interaction with membrane-mimicking surfaces, respectively. Most important, the modifications conserved the non-membrane permeabilising activity of cWFW against bacteria.
